# CXCR4 Regulates Temporal Differentiation via PRC1 Complex in Organogenesis of Epithelial Glands

**DOI:** 10.3390/ijms22020619

**Published:** 2021-01-10

**Authors:** Junchul Kim, Sang-Woo Lee, Kyungpyo Park

**Affiliations:** Department of Physiology, School of Dentistry and Dental Research Institute, Seoul National University, Seoul 110-749, Korea; jckim1@snu.ac.kr (J.K.); goodman23@snu.ac.kr (S.-W.L.)

**Keywords:** CXCR4, epithelial gland, embryonic submandibular gland, differentiation, organogenesis, polycomb repressive complex (PRC), epigenetic modulation

## Abstract

CXC-chemokine receptor type 4 (CXCR4), a 7-transmembrane receptor family member, displays multifaceted roles, participating in immune cell migration, angiogenesis, and even adipocyte metabolism. However, the activity of such a ubiquitously expressed receptor in epithelial gland organogenesis has not yet been fully explored. To investigate the relationship between CXCL12/CXCR4 signaling and embryonic glandular organogenesis, we used an ex vivo culture system with live imaging and RNA sequencing to elucidate the transcriptome and protein-level signatures of AMD3100, a potent abrogating reagent of the CXCR4-CXCL12 axis, imprinted on the developing organs. Immunostaining results showed that CXCR4 was highly expressed in embryonic submandibular gland, lung, and pancreas, especially at the periphery of end buds containing numerous embryonic stem/progenitor cells. Despite no significant increase in apoptosis, AMD3100-treated epithelial organs showed a retarded growth with significantly slower branching and expansion. Further analyses with submandibular glands revealed that such responses resulted from the AMD3100-induced precocious differentiation of embryonic epithelial cells, losing mitotic activity. RNA sequencing analysis revealed that inhibition of CXCR4 significantly down-regulated polycomb repressive complex (PRC) components, known as regulators of DNA methylation. Treatment with PRC inhibitor recapitulated the AMD3100-induced precocious differentiation. Our results indicate that the epigenetic modulation by the PRC-CXCR12/CXCR4 signaling axis is crucial for the spatiotemporal regulation of proliferation and differentiation of embryonic epithelial cells during embryonic glandular organogenesis.

## 1. Introduction

CXC-chemokine receptor type 4 (CXCR4) and its ligand, CXCL12 or SDF-1, have been investigated largely in immunology and found fame for their critical function in systemic homeostasis as the major cell migration-regulating axis [1]. Nevertheless, the roles of this axis have found no boundary, even to date. The finding that CXCR4, along with CCR5, is used by human immunodeficiency virus strains for cell entry led to the groundbreaking discovery of non-chemotactic functions of chemokine receptors [2]. A recent study showed that the CXCR4-CXCL12 axis also governs metabolism pathways where CXCL12 activated brown adipose tissue to stabilize metabolic homeostasis in mice [3]. Such flexibility is largely contributed by the stimulation of diverse G-protein-dependent and -independent pathways and the notable expression mechanism of CXCR4 characterized by agile endocytosis and recycling patterns. The cellular CXCR4 proteins, which are predominantly localized in intracellular cytoplasm, are partially-to-mostly expressed at the surface membrane upon stimulation by CXCL12 [4].

The role of this versatile receptor in development is not yet fully understood. Mutant embryos lacking CXCR4 do not survive birth, indicating its critical role in development [5]. Research on the function of the CXCR4-CXCL12 axis in organ development has shown that CXCR4 indirectly influences pancreatic development by inducing migration of the angioblasts [6]. In addition, knockdown of CXCR4 severely impairs angiogenesis of human vascular endothelial cells via the MAPK/ERK, PI3K/AKT, and Wnt/β-catenin pathways [7]. Other studies revealed the requisite function of CXCR4 in the development of renal and gastrointestinal tract vasculature [8,9]. CXCR4 or CXCL12 knockout models developed vital defects in cardiac myocytes and intestinal epithelium [10,11]. However, the role of CXCR4 during the organogenesis of epithelial organs, such as salivary glands, has not yet been elucidated.

Embryonic organogenesis of salivary glands involves a lineage specification process in which cells in the ectoderm or endoderm and mesoderm undergo differentiation at approximately embryonic day (E) 8 to form epithelium and mesenchyme, respectively [12]. The overlying mesenchyme then signals the epithelial placode to extend an initial bud at E9.5 and initiate branching morphogenesis [13]. Mesenchyme-induced signal transduction is crucial in epithelial growth as the ablation of mesenchymal factors causes growth arrest or retardation at the earliest stages of glandular organogenesis [14]. Various signaling pathways govern this complex process, including Sox transcription factors, Wnt signaling, and several kinds of growth factors, such as fibroblast growth factors and epidermal growth factors [15,16,17,18]. Most of these factors share a common purpose, to retain or confer hierarchical fate determination, the former by renewing stemness and the latter by inducing differentiation. Understanding such fate-determining factors in stem/progenitor cells enabled the generation of glandular organoids of the lung, pancreas, kidney, and salivary gland via manipulating the medium or extracellular matrix constituents [19,20,21,22].

In developmental organogenesis, the differentiation process initiated by numerous molecules is finalized through specific clusters of proteins regulating DNA methylation and subsequent condensation. The most-studied complex mediating genome-level changes during developmental stages is the polycomb repressive complex (PRC). The PRC is a large multiprotein complex involved in retaining the epigenetic memory of cell identity [23]. Cell fate determination and the maintenance of such fate are critical in the development and differentiation processes at both embryonic and adult stages. PRC, constituting major domains for methyl recognition, DNA binding, and ubiquitin-ligation, plays a crucial role in regulating the temporal transcription of developmental genes during embryonic stages [24,25]. However, no prior study has elucidated the functions of the PRC in salivary gland organogenesis. Hence, we adopted an embryonic submandibular gland (eSMG) ex vivo culture system, which provides access to visually apparent budding and clefting procedures, rendering their accurate quantification over the range E13–E16 [26]. Moreover, eSMGs have two distinctive terminal differentiation pathways, acinar and ductal lineages, and thus enable robust yet decisive recognition of temporal differentiation profiles with immunofluorescence.

In this study, we demonstrate that CXCL12/CXCR4 signaling spatiotemporally coordinates epithelial stem/progenitor cell expansion and differentiation in the organogenesis of salivary glands by regulating the expression of PRC1 components. Inhibition of the CXCR4-CXCL12 axis upon treatment with the CXCR4 antagonist AMD3100 induced retarded branching morphogenesis of eSMGs yet retained viability of the cells. Accordingly, expression of the proliferation marker Ki67 was largely abated in AMD3100-treated eSMGs. RNA sequencing data of AMD3100-treated eSMGs revealed consistent up-regulation of differentiation marker genes, accompanied by suppression of proliferation-related pathways and significant down-regulation of PRC1-comprising genes, including *chromobox homolog 8* (*Cbx8*). Lastly, we found that PRC activity suppressed by either CXCR4 inhibition or treatment with an inhibitor of PRC induced DNA demethylation, causing precocious differentiation of acinar and ductal cells. Here, we provide the evidence for CXCL12/CXCR4 signaling-mediated regulation of epigenetic patterning in glandular organogenesis.

## 2. Results

### 2.1. Spatiotemporal Dynamics of CXCR4 and CXCL12 Expression during Branching Morphogenesis of Epithelial Organs

**Figure 1:**
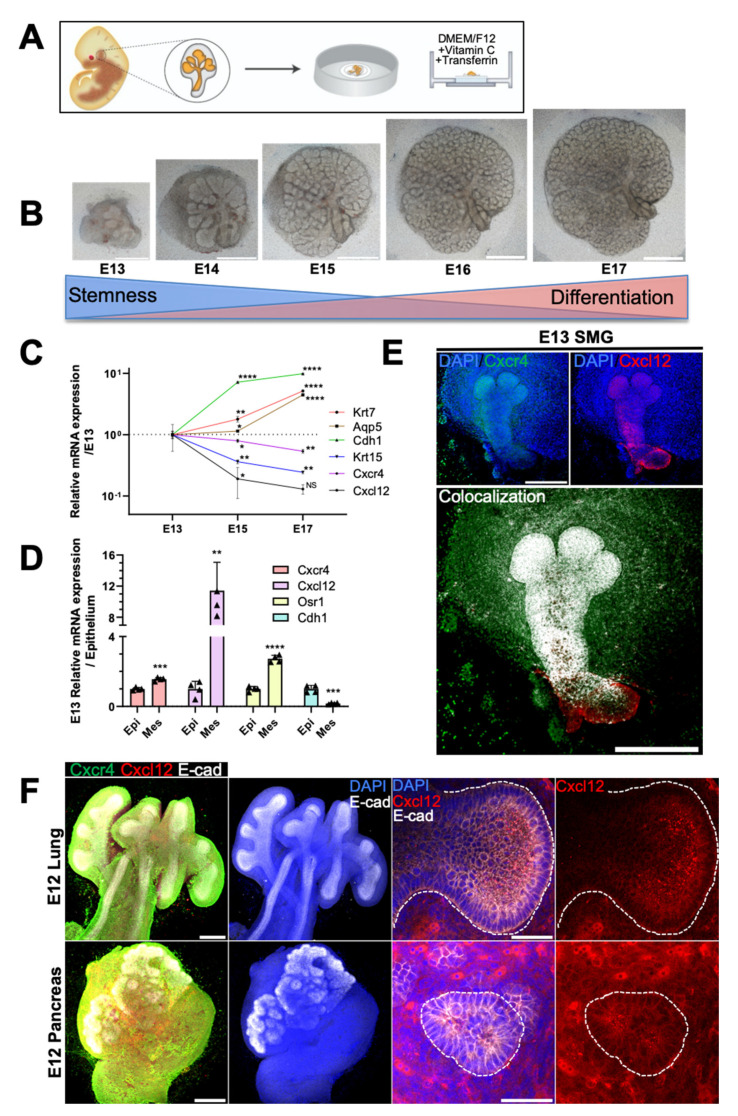
Spatiotemporal expression of CXC-chemokine receptor 4 (CXCR4) and its ligand, CXCL12, and developmental genes in embryonic organs. (**A**) Schematic diagram of embryonic submandibular gland (eSMG) isolation and ex vivo culture. (**B**) Ex vivo branching morphogenesis of eSMGs from embryonic day (E) 13 to 17, showing epithelial growth and retraction of mesenchyme. Scale bars: 500 µm. (**C**) Temporal mRNA expression patterns of *keratin 7* (*Krt7*), *aquaporin 5* (*Aqp5*), *e-cadherin* (*Cdh1*), *Krt15*, *Cxcr4*, and *Cxcl12* were measured from E13 to E17 by qPCR (*n* = 3). (**D**) Epithelial (Epi) and mesenchymal (Mes) expression of *Cxcr4*, *Cxcl12*, *odd-skipped related transcription factor 1* (*Osr1*), and *Cdh1* were quantified by qPCR at E13. The comparative *C_t_* values are expressed as fold increase relative to the epithelium (*n* = 3). (**E**) Representative images showing expression of CXCR4 and CXCL12 in eSMG (upper) and their colocalization (lower) (*n* = 3, scale bar: 500 µm). (**F**) Representative immunofluorescence images of CXCR4 and CXCL12 expression in E12 embryonic lung and pancreas (*n* = 4); whole view (left two panels; scale bar: 500 µm) and magnified lumen structures (right two panels; scale bar: 50 µm). Data are presented as the mean ± SEM; * *p* < 0.05, ** *p* < 0.01, *** *p* < 0.001, **** *p* < 0.0001, NS: not significant; two-way ANOVA (**C**); *t*-test (**D**).

To determine the spatiotemporal dynamics of CXCR4 and CXCL12 expression during murine eSMG branching morphogenesis, we adopted and followed an ex vivo culture method established by Knox et al. [27] (Figure 1A). Accordingly, eSMGs isolated at E13 were cultured for four days to observe branching morphogenesis from E13 to E17. Extensive epithelial branching and a gradual decrease in the mesenchyme occurred simultaneously during glandular organogenesis (Figure 1B). Embryonic glandular growth refers to the complex spatiotemporal-directed expression of extrinsic and intrinsic signals that drives the differentiation of epithelial stem/progenitor cells. Therefore, we monitored the temporal mRNA expression patterns of *Cxcr4* and *Cxcl12* from E13 to E17 and compared them with various progenitor/differentiated cell markers. *Cxcr4* and *Cxcl12* expression gradually decreased from E13 to E17, and a reciprocal temporal expression pattern was detected for *keratin 15* (*Krt15*), an epithelial stem cell marker (Figure 1C). By contrast, the expression levels of differentiation markers *aquaporin 5* (*Aqp5*; a marker for fully differentiated acinar cells), *Krt7* (a marker for fully differentiated ductal cells), and *e-cadherin* (*Cdh1*; a general epithelial marker) increased from E13 to E17, showing an inverse temporal relationship with *Cxcr4* and *Cxcl12* expression (Figure 1C).

Next, we determined the localization of CXCR4 and CXCL12 expression in E13 eSMGs. Epithelial rudiments and mesenchyme were separated, and the mRNA levels of *Cxcr4* and *Cxcl12* in each tissue were quantified. Separated epithelium and mesenchyme were verified with *Cdh1* as an epithelial marker and *odd-skipped related transcription factor 1* (*Osr1*) as a mesenchyme marker, respectively (Figure 1D). The mRNA expression level of *Cxcr4* was approximately two-fold higher in mesenchyme than in epithelium but a corresponding 11-fold higher for *Cxcl12* (Figure 1D). The immunofluorescence results indicated that CXCR4 was ubiquitously expressed in the epithelium and mesenchyme, with relatively stronger expression observed in the end buds and periphery of the main duct (Figure 1E, upper-left panel). CXCL12 was abundantly expressed throughout the epithelium, including the gland stem region (Figure 1E, upper-right panel). Merging the two images revealed a strong colocalized expression of CXCR4 and CXCL12 throughout the epithelial region (Figure 1E, lower panel). The periphery of the end bud in the early stage (E13–E14) of salivary gland organogenesis consists of highly mitotic embryonic stem/progenitor cells (ESCs) crucial for the propagation of branching morphogenesis [27,28,29]. Considering that CXCR4 expression was higher at the bud periphery than at the center (Figure 1E, upper-left panel), we hypothesized that CXCR4 is possibly involved in the proliferation and differentiation of such ESCs. Furthermore, we found that CXCR4 and CXCL12 were also robustly expressed in other branching epithelial organs, including the lung and pancreas, suggesting that the CXCR4-CXCL12 axis could be functionally involved in the branching morphogenesis of various epithelial organs (Figure 1F).

### 2.2. AMD3100 Perturbs Glandular Organogenesis without Apoptosis

**Figure 2:**
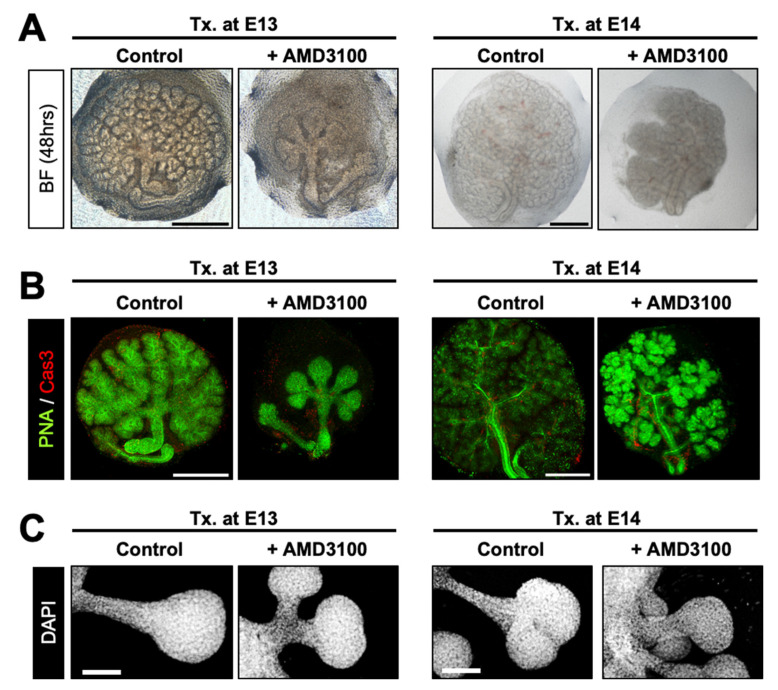
AMD3100-induced suppression of glandular organogenesis without cell death. (**A**) Representative bright-field images of eSMGs treated with AMD3100 at E13 (left column) and E14 (right column), respectively. Images were taken at 48 h after the treatment (*n* = 4, scale bar: 500 µm). (**B**) Immunostaining results of peanut lectin agglutinin (PNA, green) and cleaved caspase-3 (Cas3, red) of eSMGs treated with AMD3100 at E13 (left column) and E14 (right column), respectively. Images were taken at 48 h after the treatment (*n* = 4, scale bar: 500 µm). (**C**) The epithelial rudiments of eSMGs were cultured and treated with AMD3100 at E13 (left column) and E14 (right column), respectively. DAPI in gray. Images were taken at 48 h after the treatment (*n* = 4, scale bar: 100 µm).

To elucidate the roles of CXCR4 during branching morphogenesis, we performed loss-of-function experiments by treating ex vivo cultured eSMGs with the highly selective CXCR4 inhibitor, AMD3100. This symmetric bicyclam, used throughout this study to inhibit CXCR4, is not toxic to host cells even at 500 µM, attesting to both the effectiveness and safety of the molecule [30]. When E13 eSMGs were treated with 50 µM AMD3100, further branching process was completely abolished. Thus, bud size and number were unchanged even after 48 h of culture (1.5-fold change [FC] in bud number) (Figure 2A, left panel, and Figure S1A). By contrast, the 48 h-cultured control eSMGs showed extensive branching (23 FC in bud number) and enlarged bud size (Figure 2A, left panel, and Figure S1A). Interestingly, AMD3100-treated E14 eSMGs displayed propagation of branching morphogenesis (65 FC in bud number), but it was significantly inhibited compared with control eSMGs (210 FC in bud number) (Figure 2A, right panel, and Figure S1C). To determine whether such retarded branching by AMD3100 was due to cell death, we immunostained cleaved caspase-3, a representative marker of apoptosis. Cleaved caspase-3 was barely detected in the epithelium (stained with peanut lectin agglutinin) of both control and AMD3100-treated eSMGs, indicating that AMD3100-induced suppression of branching morphogenesis was not a result of apoptosis (Figure 2B and Figure S1B,D). Similar phenomena were observed in AMD3100-treated embryonic lung and pancreas; AMD3100 significantly inhibited branching morphogenesis without inducing apoptosis (Figure S2A–D). In addition, malformations in bronchial buds and pancreatic acini were observed in the AMD3100-treated groups (Figure S2A,B). We treated E13 and E14 eSMG epithelial rudiments with AMD3100 after mesenchyme removal to further confirm these results. Under this condition, mesenchymal factors can be minimized, and the observation of minuscule structures, such as ducts or clefts, can be improved. Epithelial rudiments treated with AMD3100 showed significantly shorter ducts than the control groups, regardless of the treatment timing (Figure 2C and Figure S1E,F).

In summary, we found that inhibition of CXCR4 resulted in significant retardation of branching morphogenesis without apoptosis, and the degree of retardation depended on the timing of CXCR4 inhibition. Based on these results, we hypothesized that the AMD3100-induced retardation of branching morphogenesis was due to the precocious differentiation of stem/progenitor cells because E13 eSMGs contain less differentiated stem/progenitor cells than E14 eSMGs.

### 2.3. AMD3100 Opposingly Regulates Proliferation and Differentiation in Developing eSMGs

**Figure 3:**
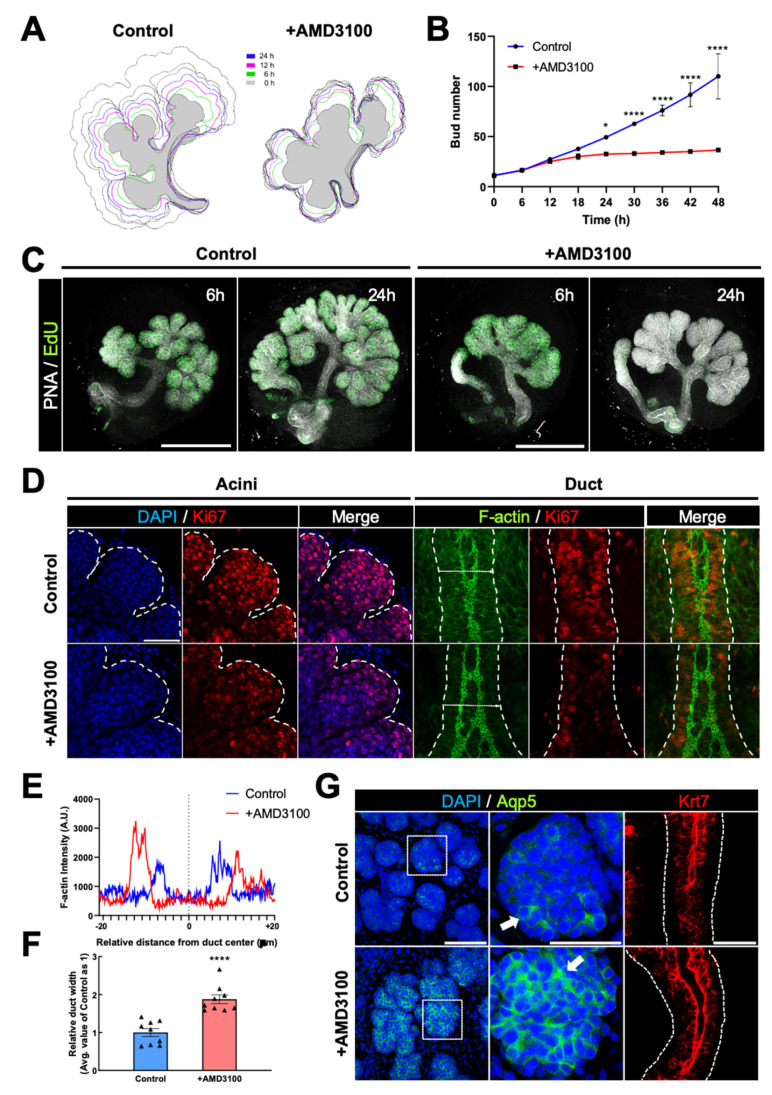
AMD3100-induced precocious differentiation of epithelial cells. (**A**,**B**) Representative contour tracing (**A**) and bud number changes (**B**) of control and AMD3100-treated eSMGs during 48 h at 6-h intervals (*n* = 3). (**C**) EdU staining results at 6 and 24 h after AMD3100 treatment. EdU in green and PNA in gray (*n* = 4, scale bar: 500 µm). (**D**) Immunostaining results of Ki67 (red) and F-actin (green) in acini and duct of eSMGs 24 h after AMD3100 treatment. Morphologies of acinar buds and duct cells are outlined with white dotted lines. Scale bar: 50 µm. (**E**) Duct widths of control and AMD3100-treated eSMGs were visualized via F-actin-based intensity profiles of horizontal sectioning of ducts 24 h after the treatment. (**F**) Duct widths of control and AMD3100-treated eSMGs were quantified 24 h after the treatment (*n* = 9). (**G**) Immunostaining results of AQP5 (green) and KRT7 (red). Magnified regions of acinar buds are marked with white dotted squares. The white arrows (middle panels) indicate areas with the highest AQP5 expression (*n* = 4, scale bar: left, 100 µm; middle and right, 50 µm). Data are presented as the mean ± SEM; * *p* < 0.05, **** *p* < 0.0001; *t*-test.

To prove the hypothesis above, we decided to monitor the molecular dynamics of eSMGs treated with AMD3100 at E14, rather than at E13, because the differentiation of acinar and ductal cells can be observed and quantified by temporal expression of AQP5 and KRT7 at > E14, respectively [27]. First, to improve the understanding of the temporal dynamics of CXCR4-induced retardation in branching morphogenesis, the eSMGs were imaged in real-time. Based on the contour tracing and bud number count, we noticed that the retardation of branching morphogenesis was initiated 12 h after AMD3100 treatment and became statistically evident at 24 h (Figure 3A,B). These results suggest that the retardation accompanies changes in global gene expression.

There is a widely-accepted consensus that as stem cells differentiate, their mitotic rate usually decreases [31]. Therefore, if the AMD3100-induced retardation of branching morphogenesis is because of the precocious differentiation of ESCs, AMD3100-treated eSMGs should show decreased mitotic activity. As expected, significantly decreased expression of Ki67 and EdU signals, representative markers of proliferation, were observed in both acinar and ductal cells of AMD3100-treated eSMGs (Figure 3C,D and Figure S3A,B), supporting the apoptosis-free retardation of branching morphogenesis and shortened duct length observed in AMD3100-treated eSMGs (Figure 2). In addition, F-actin staining images of duct structure revealed that the formation of lumenized ducts progressed more extensively in AMD3100-treated groups than in control groups (Figure 3E,F). Considering that duct lumenization is initiated at E14 and further progressed by expanding the luminal space as the branching morphogenesis propagates [32], more expanded lumen in AMD3100-treated groups indicates that the duct lumenization is initiated earlier than in control groups.

Finally, we compared AQP5 (an acinar differentiation marker) and KRT7 (a ductal differentiation marker) expression levels between control and AMD3100-treated groups to acquire direct evidence that inhibition of CXCR4 induces precocious differentiation of epithelial stem/progenitor cells. As expected, mRNA and protein expressions of AQP5 and KRT7 were significantly higher in AMD3100-treated groups than in control groups, indicating that precocious differentiation of acini and duct had occurred as a result of CXCR4 inhibition (Figure 3G and Figure S3C,D).

### 2.4. AMD3100 Alters Expressions of Developmental and PRC1-Comprising Genes in eSMGs

**Figure 4:**
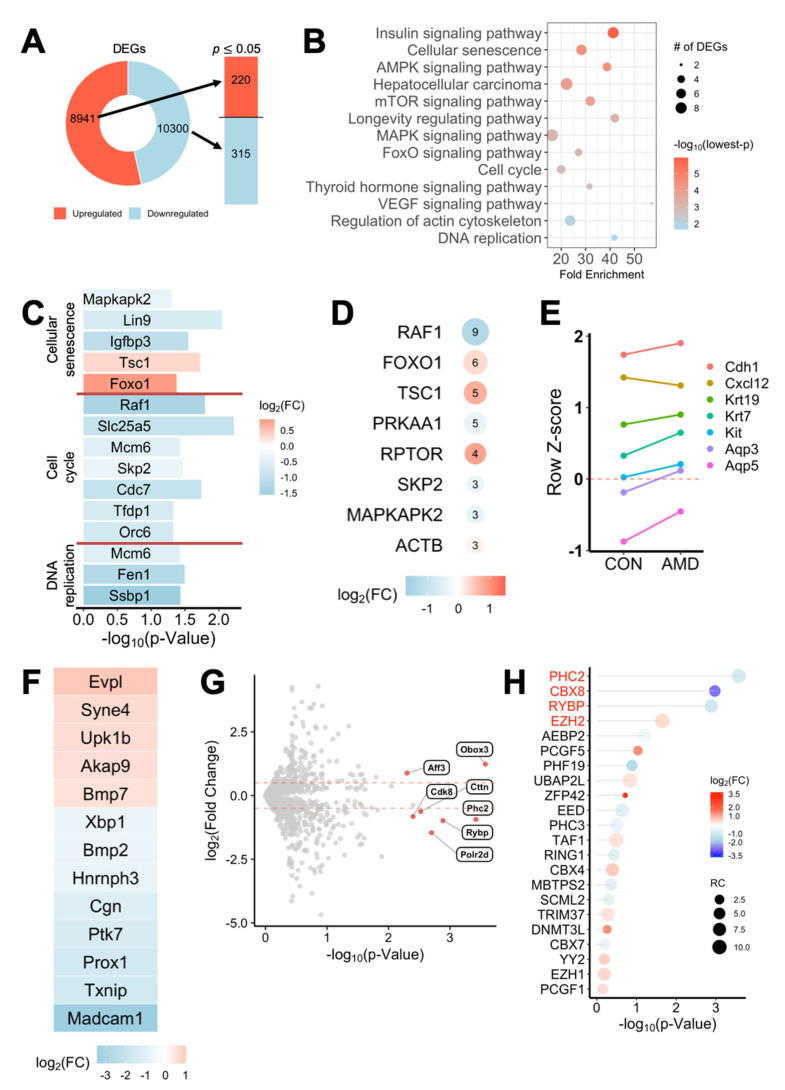
AMD3100-induced transcriptional changes in developmental and polycomb repressive complex 1 (PRC1) genes. (**A**) 3′ mRNA sequencing was performed on control and AMD3100-treated E14 eSMGs at 6 h. Among 8941 up-regulated and 10,300 down-regulated genes in the treated group, a total of 535 differentially expressed genes (DEGs) with *p* < 0.05 and |log2 fold change (FC)| ≥ 1 were found; 220 up-regulated and 315 down-regulated. (**B**) Kyoto Encyclopedia of Genes and Genomes (KEGG) pathway enrichment analysis of DEGs. Significantly enriched pathways are revealed by enrichR. (**C**) DEGs in KEGG pathways cellular senescence, cell cycle, and DNA replication are shown with log2FC and *p*-values. (**D**) The most frequently occurring genes in significantly enriched pathways are listed with log2FC and their number of occurrences. (**E**) Read count *z*-scores of epithelial differentiation marker genes consistently increased in AMD3100-treated eSMGs, contrasting to the decrease in *Cxcl12*. (**F**) DEGs were filtered out from the Gene Ontology (GO) gene set GO:0030855—epithelial cell differentiation—and the inclusive DEGs are listed. (**G**) Currently-known murine transcription factors (1475 total) were extracted from PantherDB to filter the DEGs. Transcription factor genes with *p* ≤ 0.01 and |log2FC| ≥ 0.5 are in red. (**H**) Genes comprising PRC1 complex are listed with log2FC and *p*-values. Names in red indicate genes with *p* < 0.05.

In light of the pivotal point at which AMD3100-induced changes were discovered in the previous assays, we performed 3′ mRNA sequencing on control and AMD3100-treated eSMGs to determine how CXCR4 regulates epithelial differentiation during organogenesis. As a result, among 8941 up-regulated and 10,300 down-regulated genes, 220 and 315 significant (FC > 1.5, *p* < 0.05) genes were extracted for differential analysis, respectively (Figure 4A). The clustering heatmap and volcano plot are shown in Figure S4A,B, respectively. *Profilin 1* (*Pfn1*) and *Cbx8* appeared among the top significant genes (FC > 2, *p* < 0.01), as evidenced in the volcano plot. Both genes were substantially down-regulated in AMD3100-treated groups, suggesting misguided cytoskeleton rearrangement and detrimental formation of PRC1, in which CBX8 plays a crucial role.

To elucidate the signaling pathways altered by AMD3100 treatment, we performed KEGG pathway enrichment analysis of the significant genes. Several pathways, such as cellular senescence, cell cycle, and DNA replication, were highly enriched (Figure 4B). The genes in these three pathways were listed, and except for *tuberous sclerosis 1* (*Tsc1*) and *forkhead box O1* (*Foxo1*) in the cellular senescence pathway, all genes were significantly down-regulated (Figure 4C). The four most enriched KEGG pathways (insulin signaling, cellular senescence, AMPK signaling, mTOR signaling) shared the *Tsc1* gene, which is known to inhibit proliferation-promoting mTOR activation (Figure S4C). To identify the gene of major influence among the enriched pathways, highly enriched gene sets were cross-analyzed to find frequently overlapping genes. Nine of the top 13 gene sets included *Raf1*, which was significantly down-regulated in AMD3100-treated eSMGs (Figure 4D).

Next, we examined the genes related to normal development and differentiation of epithelial cells. As shown in Figure 4E, except for *Cxcl12*, whose expression was on the wane, the expressions of acinar (*Aqp3* and *Aqp5*) and ductal differentiation markers (*Krt19* and *Krt7*) were increased in the AMD3100-treated group, supporting the previous results (Figure 3G). To confirm the notable changes in genes related to epithelial differentiation, we screened the whole gene set by genes included in Gene Ontology (GO) gene set GO:0030855—epithelial cell differentiation. We identified 13 out of 568 genes that were up-regulated or down-regulated by over 1.5-fold (*p* < 0.05) in the treated group (Figure 4F). The two most up-regulated genes were *envoplakin* (*Evpl*), a component of keratinocytes and whose expression is known to increase during cell differentiation, and *nesprin-4* (*Syne4*), a component of the linker of nucleoskeleton and cytoskeleton (LINC) complex, and suggested in previous studies to contribute to the secretory epithelial morphology [33,34].

We continued further analysis to reveal significant transcription factors involved in regulating the selected pathways and genes above. All currently known murine transcription factors extracted from the PantherDB database (1475 total) were analyzed against our gene set (Figure 4G). Seven transcription factors were differentially (FC > 1.5, *p* < 0.05) expressed in the treated eSMGs, and three of them were genes of PRC1. Genes comprising PRC1 (*p* < 0.05) were listed, and changes in expressions of *polyhomeotic-like protein 2* (*Phc2*), *Cbx8*, *RING1 and YY1 binding protein* (*Rybp*), and *enhancer of zeste homolog 2* (*Ezh2*) were found to be of importance (Figure 4H). The qPCR results of the key relevant genes are shown in Figure S4D.

### 2.5. PRC1 Perturbation Induces Precocious Differentiation in Acinar and Ductal Cells of eSMGs

**Figure 5:**
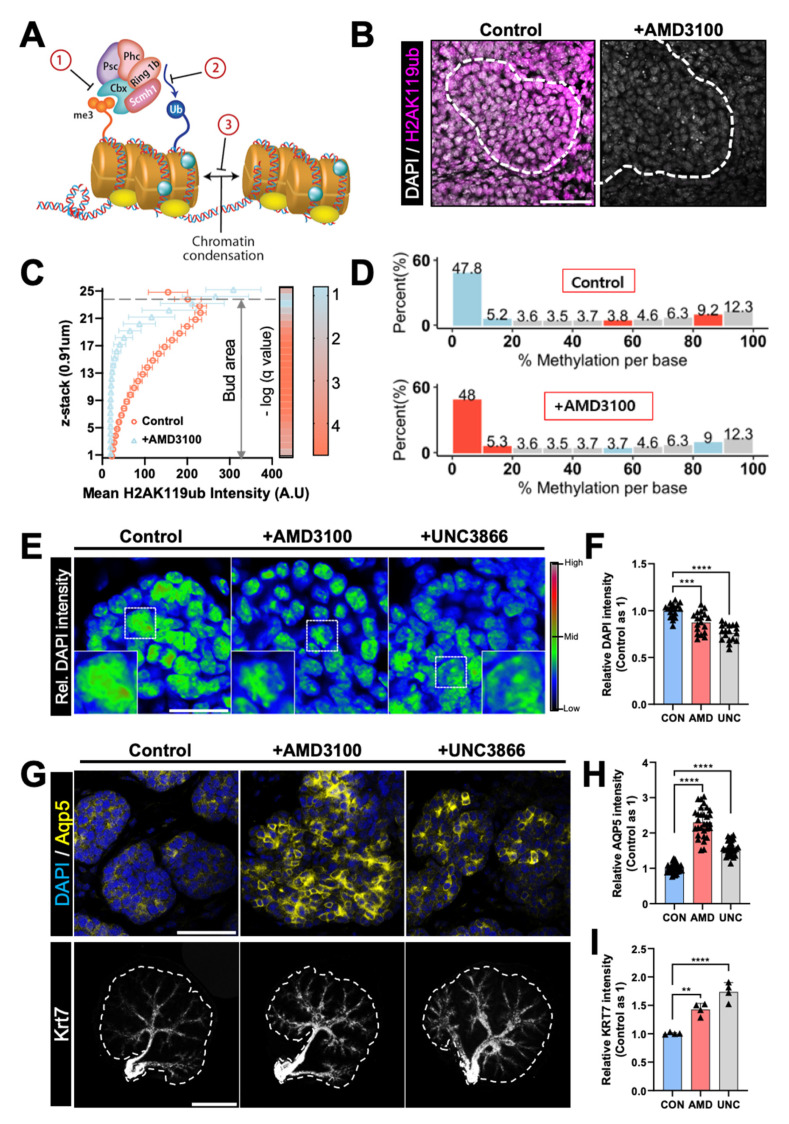
eSMGs lacking PRC1 develop precocious differentiation in acini and ducts. (**A**) Schematic diagram of AMD3100-induced effect on organogenesis. Down-regulation of PRC1 genes leads to the inability of methylation site recognition by chromobox 8 (CBX8), a polycomb group protein (**1**), resulting in failure of the ubiquitin-ligating activity of ring finger protein 1 (RING1) (**2**) and thus, disabled DNA condensation (**3**). Hence, the developmental genes are readily transcribed by RNA polymerase II. (**B**) Representative images showing ubiquitin expression at Lys119 of histone 2A (H2AK119ub) in control and AMD3100-treated eSMGs 24 h after the treatment (*n* = 4, scale bar: 50 µm). (**C**) Mean fluorescence intensities of H2AK119ub in epithelial buds per *z*-stack of 0.91 µm (25 stacks per end bud). False discovery rate (FDR) *q*-values of the differences in H2AK119ub expression at each of the 25 stacks are shown in the heat map, in which 23 out of 25 stacks marked a significant difference between control and AMD3100-treated eSMGs. -log(*q*-value) > 1.3 is considered significant (*q* < 0.05; *t*-test with 5% FDR correction). The heat map (right) shows the color profile (*n* = 4). (**D**) Methyl-seq results from control and AMD3100-treated eSMGs. Ten eSMGs per group were homogenized and analyzed. (**E**) Relative DAPI intensities of end buds in control, AMD3100-, and UNC3866-treated groups are expressed as a colored heat map (0–65,535 gray-value). Magnified regions are marked with white dotted squares (*n* = 4, scale bar: 20 µm). (**F**) Quantification of DAPI intensities in nuclei of control (CON), AMD3100 (AMD)-, and UNC3866 (UNC)-treated eSMGs (*n* = 18). (**G**) Immunostaining results of AQP5 (yellow) and KRT7 (gray) in eSMGs treated with AMD3100 or UNC3866. eSMGs were fixed and immunostained 24 h after the treatment (*n* = 4, scale bar: upper panel, 50 µm; lower panel, 500 µm). (**H**) Quantification of AQP5 intensities in acinar buds of eSMGs treated with AMD3100 (AMD) or UNC3866 (UNC) (*n* = 30). (**I**) Quantification of KRT7 intensities in ducts of eSMGs treated with AMD3100 (AMD) or UNC3866 (UNC) (*n* = 4). Data are presented as the mean ± SEM; ** *p* < 0.01, *** *p* < 0.001, **** *p* < 0.0001; one-way ANOVA.

PRC1 has been extensively studied for its role in DNA methylation of developmental genes and chromatin condensation [35]. Briefly, PRC1 finalizes the silencing of genes that maintain ESC identity by recognizing trimethylated histone H3 lysine 27 (H3K27me3) and tagging a ubiquitin at Lys119 of histone 2A (H2AK119ub) [36] (Figure 5A). H2AK119ub+ cells can be considered as relatively more methylated because H2AK119ub stabilizes the activities of PRC1 and PRC2, promoting DNA condensation and thus ensuring gene silencing [37]. In developing eSMGs, H2AK119ub+ cells were observed in the mesenchyme and periphery of end buds containing proliferative and temporally undifferentiated stem/progenitor cells (Figure 5B, left panel). However, following treatment with AMD3100, the expression of H2AK119ub virtually disappeared, suggesting a possibility of intense demethylation (Figure 5B, right panel).

To compare the three-dimensional expression patterns of H2AK119ub in control and AMD3100-treated groups, we analyzed a total of 25 *z*-stack (referring to the *z*-axis) immunofluorescence images (0.91 μm per stack) for each epithelial bud. The mean fluorescence intensities of *z*-stack images from the top (25th stack) to the bottom (first stack) of end buds were quantified; the top region in contact with the mesenchyme and the bottom part attached to the filter membrane (Figure 5C). In control eSMGs, H2AK119ub expression was highest at the top of the bud region directly in contact with the mesenchyme, then progressively decreased toward the bottom. However, AMD3100-treated eSMGs showed a relatively precipitous decrease in H2AK119ub expression along the *z*-axis. Statistical analysis revealed that except for the 23rd and 24th stacks, H2AK119ub expression within the entire *z*-stacks was significantly lower in AMD3100-treated groups than in control groups (Figure 5C, right heat map). These results indicate that the AMD3100-induced decrease in H2AK119 ubiquitination occurs throughout the entire epithelial bud area, suggesting that the degree of developmental gene silencing might have been decreased. When the PRC1-mediated ubiquitin tagging fails to occur, the subsequent disruption of methylation and euchromatin condensation can be observed by the reduced intensity of DAPI staining in the nucleus [38]. As expected, DAPI signals of AMD3100-treated eSMGs were reduced significantly compared with the control group (Figure S5A).

To further confirm the change in DNA methylation status, we performed Methyl-seq for control and AMD3100-treated eSMGs 6 h after the treatment. The ratios of methylated to total cytosines in a CpG context were 39.9% and 39.7% in control and AMD3100-treated eSMGs, respectively. In AMD3100-treated eSMGs, however, the methylation degree of highly methylated loci (>80% methylation) was reduced by 1% compared with control groups, while a 1% increase was observed in loci with < 20% methylation (Figure 5D). Over 30 million cytosines, in a CpG context, were methylated in both groups, and a 1% decrease of highly methylated regions in 6 h revealed the instantaneous demethylating effect of AMD3100 treatment.

Here, we hypothesized that the AMD3100-induced decrease in PRC1 component expressions would fail to entail ubiquitination of H2AK119 in DNA, disrupting temporal methylation of late-stage differentiation marker genes. To determine whether the down-regulation or inhibition of PRC1 can recapitulate the precocious differentiation of acini and duct induced by CXCR4 inhibition, we used 20 µM UNC3866, a potent antagonist of the methyl-lysine reading function of the CBX proteins [39]. AMD3100- and UNC3866-treated eSMGs showed significantly reduced DAPI signal intensities, that is, less condensed DNA than control eSMGs (Figure 5E,F). There was no significant difference in the DAPI signal intensities between AMD3100- and UNC3866-treated eSMGs (Figure 5E,F). More importantly, treatment with UNC3866 recapitulated AMD3100-induced precocious differentiation of acini and duct in developing eSMGs. When E14 eSMGs were treated with AMD3100 and UNC3866, a rapid increase in AQP5 expression was observed within 24 h, while only a weak expression of AQP5 was detected in control eSMGs (Figure 5G, upper panel, and 5H). Similarly, compared with control eSMGs, significantly elevated expression of KRT7 was observed in AMD3100- or UNC3866-treated eSMGs (Figure 5G, lower panel, and 5I). In addition, the subduct lengths were also decreased in UNC3866-treated eSMGs (Figure S5B), recapitulating the AMD3100-induced duct length decrement observed in Figure 2C. Together, these results suggest that abnormal demethylation and transcription of developmental genes caused by down-regulation of PRC1 components are the key mechanisms for AMD3100-induced precocious differentiation of ductal and acinar progenitor cells.

## 3. Discussion

Our results elucidated the mechanistic link between CXCR4 and branching morphogenesis of embryonic salivary glands. We revealed that the expression of CXCR4 is spatiotemporally regulated, and this dynamicity of CXCR4 expression orchestrates the timing of proliferation and differentiation during branching morphogenesis of eSMGs. In addition, our mRNA sequencing results suggested that CXCR4 regulates a significant number of genes. In particular, the expressions of PRC genes *Phc2*, *Cbx8*, *Rybp*, and *Ezh2*, were the most significantly altered by pharmacological inhibition of CXCR4. We also provided evidence that timely regulated DNA methylation via dynamicity of the PRC-CXCR4 axis is crucial for branching morphogenesis of eSMGs.

Although AMD3100, the agent used throughout this study, was found to be abrogating both CXCR4 and CXCR7 by interaction with CXCL12 [40], Heinrich et al. showed that CXCR7 and CXCR4 mediate different pathways and that initiation of the K-Ras pathway only follows activation of the CXCR4-CXCL12 axis [41]. Ras regulates the upstream pathways of activation of the PRC1-Cyclin D1 complex and trimethylation of H3K27me3 [42,43], providing the rationale for the AMD3100-induced down-regulation of genes in the Ras-Raf signaling pathway. Another reason for using AMD3100 was that it is a commercial pharmaceutical reagent (Plerixafor), introduced to induce migration of hematopoietic stem cells from bone marrow to peripheral blood, providing relatively safer access to clinical applications than other molecules [30].

**Figure 6:**
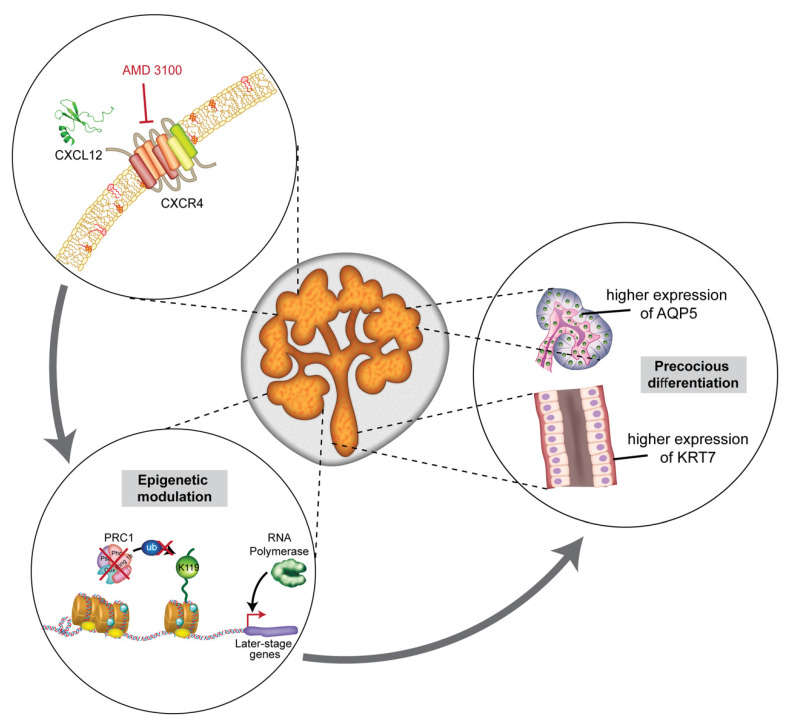
Schematic for AMD3100-induced epigenetic modulation and precocious differentiation in eSMGs. The treatment of eSMGs with AMD3100 is followed by down-regulation of PRC1 components, leading to the absence of ubiquitin-ligating activity on H2AK119, which, in turn, deprives the DNA of its condensing function. Such epigenetic modulation confers de novo or stronger transcription of later-stage developmental genes to eSMGs, promoting precocious differentiation in acini and ducts.

Based on our results, we propose a hypothetical model for salivary gland organogenesis. First, high-level expression of CXCR4 and CXCL12 at the early stage (E13–E14) of branching morphogenesis maintains high-level DNA methylation that supports pluripotency and proliferation of embryonic progenitor cells sufficiently to induce organization of the size and structure of the organ. Second, as the branching morphogenesis propagates, CXCR4 expression decreases with perishing mesenchyme, leading to the production of vast quantities of CXCL12. In turn, the influence of CXCR4 wanes. As a result, the expression level of PRC components decreases, and subsequent DNA dispersal and enhanced transcription of developmental genes occur to promote acinar and ductal differentiation at the late stage of organogenesis (Figure 6). However, because eSMGs contain multiple heterogeneous cell populations, including acinar, ductal, stem/progenitor, parasympathetic ganglion, myoepithelial, and vascular endothelial cells, the effects of the CXCR4 inhibitor must have varied by cell type [44]. These various cell populations interact with each other for proper branching morphogenesis, and so cell type-specific effects induced by CXCR4 inhibition may have influenced our results. Although it is extremely difficult to examine the cell-type-specific roles of CXCR4 independently, such effort will greatly improve insight into CXCL12/CXCR4 signaling during salivary gland branching morphogenesis.

We found that the branching morphogenesis of other epithelial organs, such as the lung and pancreas, is also affected by CXCR4 activity but could not elucidate the details of the underlying mechanism’s details. Although we speculate that other branching organs may share the same or similar mechanism to that found in salivary glands, further organ-specific mechanism studies are required for a definitive result. Another limitation of our study is that it relied largely on pharmacological perturbation-based loss-of-function experiments. Although it is remarkably challenging to deliver plasmid, siRNA, and the CRISPR/Cas9 system to eSMG epithelium with acceptable efficiency, genetic perturbation of CXCR4 or CXCL12 should be performed to fully elucidate the roles of CXCR4 in the branching morphogenesis of developing epithelial organs.

Nevertheless, several studies support our results on the relationship between PRC functions and stem/progenitor cell pluripotency. PRC1 proteins secure ESC identity maintenance by repressing the transcription of crucial developmental genes [45]. Specifically, the DNA binding function of CBX family proteins, Ring1A/B-mediated H2A mono-ubiquitylation of H2A at Lys119, and recognition of PRC2-mediated H3K27me3 by PHC2 are some of the major functions and related constituents of the complex. CBX family proteins are canonical components in PRC1, responsible for targeting PRC1 to the chromatin [46]. CBX8, whose gene was significantly down-regulated in our study, and H3K27me3, physically interact via their chromodomains [47]. In turn, RING1A, another component of the PRC1 complex, attaches a ubiquitin at Lys119 of histone 2A (H2AK119) to further induce DNA condensation, disabling the transcription activity of RNA polymerase II on developmental genes. A deficit of any comprising proteins from the structure leads to severe function loss of the whole complex, leading to chromatin dispersal and DNA demethylation. Previous work on the roles of PHC2 in the mobilization of hematopoietic progenitor cells showed that PHC2, as a component of canonical PRC1, regulated H2AK119ub to repress vascular cell adhesion molecule-1 (Vcam1) expression [48]. Research on the depletion of PRC1 proteins EED or RING1B was followed by increased expression of differentiation markers in mouse ESCs [49]. Considering that ESCs have higher methylation rates than differentiated cells [50], such a global demethylation effect of CXCR4 inhibition would impose a critical defect on early embryonic development, including gastrulation, possibly contributing to prenatal death of CXCR4 knockout embryos. In addition, DNA methylation is a major spatiotemporal modulator of both pluripotency and differentiation during organogenesis [51]. In salivary gland organogenesis, genes related to the proliferation of c-Kit+-embryonic progenitor cells are epigenetically regulated by microRNAs in exosomes secreted from the mesenchyme [52]. In addition to the progenitor cell expansion, epigenetic modification is also involved in mapping differentiation patterns in salivary glands. Shin et al. reported that the mechanism for differential expression of anoctamin-1 protein, a calcium-activated chloride channel, in acini and duct is dynamic alternation in DNA methylation occurring in the acinar/ductal differentiation process during salivary gland organogenesis [53]. Our results suggest that such differentially patterned expressions of cell type-specific genes in salivary glands are possibly determined by CXCL12/CXCR4 signaling and subsequent epigenetic modifications.

It is noteworthy that the activity of PRC, reflected as H2AK119ub positivity, is high in the peripheral cell layers of epithelial end buds, while few H2AK119ub+ cells are found in inner bud cells. Cell layer-specific expression of certain genes during epithelial organ development is considered a crucial factor for morphogenesis. In salivary glands, locally confined expression of voltage-dependent calcium channels at the peripheral cell layers of the epithelial bud promotes epithelial cleft formation [54]. In prostate development, basal epithelium-specific DNA methylation of the *Cdh1* gene within the urogenital sinus epithelia is required to initiate prostatic bud formation [55]. Likewise, our results implicate a possibility that highly localized and finely tuned gene expression by DNA methylation is necessary to propagate branching morphogenesis in salivary glands. However, because the Methyl-seq performed in our study describes the methylation states of the whole eSMG, the result cannot fully reflect the methylation states of genes specifically localized at the periphery or inner buds. Reliable tissue separation techniques, such as laser microdissection, are required to accurately examine localized methylation levels of specific genes [56].

Another important aspect that should be elucidated in future studies is the mechanistic link between CXCL12/CXCR4 signaling and the expressional regulation of PRC components. The direct PRC regulation of the Ras-Raf signaling pathway was postulated, but CXCL12/CXCR4 signaling also regulates several transcription factors, including NFAT, NF-κB, Elk-1, and Egr1 [57]. Therefore, binding assays of these transcription factors and promoter regions of PRC components down-regulated by CXCR4 inhibition may further clarify how CXCR4 can regulate the expression of PRC components.

Taken together, the results of our study confirm that the epigenetic modulation by expressional regulation of PRC1 components via CXCL12/CXCR4 signaling spatiotemporally coordinates the proliferation and differentiation of developing salivary glands. Although CXCL12/CXCR4 signaling positively regulates the regeneration of multiple organs, such as the liver, lung, heart, and nervous system [58], its involvement in the context of salivary gland development has not been investigated previously. Therefore, our findings will ultimately contribute to the therapeutic approach of epithelial gland regeneration by expanding the understanding of the glandular organogenesis of epithelial organs.

## 4. Materials and Methods

### 4.1. Materials

Nuclepore^®^ polycarbonate (PC) track-etched membranes, transferrin, L-ascorbic acid, and AMD3100 were purchased from Sigma-Aldrich (St. Louis, MO, USA). UNC3866 was bought from Selleckchem (Houston, TX, USA). Dulbecco’s modified Eagle medium with F12 supplement (DMEM/F12, 1:1) was purchased along with penicillin-streptomycin from Gibco (Grand Island, NY, USA).

### 4.2. Ex Vivo Culture of eSMGs

eSMGs were collected from the submaxilla of E13 to E17 embryos and were placed on a Nuclepore^®^ PC membrane (Sigma-Aldrich, 110405). DMEM/F12 1:1 (Gibco, 21041-025) supplemented with 1% *v/v* penicillin-streptomycin (Gibco, 15140122), 150 µg/mL ascorbic acid (Sigma-Aldrich, A5960), and 50 µg/mL transferrin (Sigma-Aldrich, T8158) was used as culture medium. The eSMGs were then cultured in an incubator at 37 °C for 6 to 48 h with 5% CO_2_. AMD3100 and UNC3866 were added at 50 and 20 µM, respectively, to the medium. The protocol for the animal experiments in this study was approved by the Seoul National University Institutional Animal Care and Use Committee (approval number: SNU-190320-7).

### 4.3. Epithelial Rudiment Culture

Mesenchyme and epithelium were separated by enzymatic digestion of E13–E13.5 eSMGs with 0.5 U/mL Dispase I (Life Technologies, 17105-041; Grand Island, NY, USA) for 20 min, followed by physical separation using forceps under a stereomicroscope. Isolated epithelial rudiments were encapsulated in growth-factor reduced Matrigel (BD Biosciences, 356231; Franklin Lakes, NJ, USA) and then cultured with the culture medium (described above). 10 ng/mL EGF (R&D Systems, 236-EG; Minneapolis, MN, USA), and 100 ng/mL Fgf7 (R&D Systems, 251-KG).

### 4.4. Real-Time Live Imaging

The culture medium (as described above) was placed in the well of the SPLInsert^®^ hanging plate (SPL, 35124; Gyeonggi, Korea). The PC membranes of the inserts were carved out, and the Nuclepore^®^ PC membrane was fixed between each of the inserts and medium for stable image acquisition. Extracted eSMGs were then placed on the PC membranes and imaged in real-time for 48 h using an EVOS^®^ FL Auto 2 Imaging System (ThermoFisher, 15736152; Waltham, MA, USA).

### 4.5. Immunofluorescence Staining and Imaging

Cultured eSMGs were fixed with 4% *v*/*v* paraformaldehyde (PFA; Tech & Innovation, BPP-9004; Gangwon, Korea) at 4 °C for 20 min, then permeabilized with 0.1% *v*/*v* PBS-Triton^®^ X-100 (PBSX; Merck Millipore, 108643; Billerica, MA, USA) at room temperature (RT) for 20 min. eSMGs were blocked by incubation with 10% *v*/*v* normal donkey serum (NDS; Sigma-Aldrich, D9663), 1% *w*/*v* BSA, and 1% *v*/*v* mouse on mouse (MOM) IgG-blocking reagent (Vector Labs, MKB-2213-1; Burlingame, CA, USA) in 0.05% *v*/*v* PBS-Tween^®^ 20 (Sigma-Aldrich, P1379) at RT for 3 h. Next, eSMGs were incubated with PBSX containing 3% *v*/*v* NDS and primary antibodies (1:100) at 4 °C overnight. The following primary antibodies were used in the procedures: rat monoclonal anti-CXCR4 antibody (R&D Systems, MAB21651), rabbit monoclonal anti-KRT7 antibody (Abcam, ab181598; Cambridge, UK); rabbit monoclonal anti-CDH1 antibody (CST, 3195; Beverly, MA, USA), rabbit monoclonal anti-H2AK119ub antibody (CST, 8240); mouse monoclonal anti-CXCL12 antibody (Novus Biologicals, MAB350; Littleton, CO, USA), rabbit polyclonal anti-AQP5 antibody (Alomone Labs, AQP-005; Jerusalem, Israel), rabbit monoclonal anti-CASP3 antibody (CST, 9664), and rat monoclonal anti-KI67 antibody (ThermoFisher, 14-5698-82). The unreacted primary antibodies were washed with PBSX four times (10 min per wash) at RT. Then, eSMGs were incubated with PBSX with 3% *v*/*v* NDS, secondary antibodies (1:250), and DAPI (1:1000) at 4 °C overnight. The following secondary antibodies were used in the procedures: donkey anti-rat IgG (H+L) Alexa Fluor^®^ 488 conjugate (Invitrogen, A21208; Carlsbad, CA, USA), donkey anti-mouse IgG (H+L) Alexa Fluor^®^ 594 conjugate (Invitrogen, A21203), donkey anti-rabbit IgG (H+L) Alexa Fluor^®^ 647 conjugate (Invitrogen, A32795), phalloidin-Texas Red^®^ conjugate (ThermoFisher, T7471), and PNA lectin FITC conjugate (Sigma-Aldrich, L7381). The unbound secondary antibodies were washed with PBSX four times (10 min per wash). Each of the PC membranes with stained eSMGs was mounted on a slide and imaged using an LSM700 confocal microscope with 10×/0.45 Plan-Apochromat air or 40×/1.0 Plan-Apochromat water-immersion objectives.

### 4.6. EdU Staining

EdU staining was applied using the Click-iT^®^ EdU Alexa Fluor^®^ 594 Imaging Kit (Invitrogen, C10339) according to the manufacturer’s instructions. In eSMG culture, the medium was supplemented with 10 µM EdU and incubated at 37 °C for 30 min. eSMGs were then fixed and permeabilized as per the other immunofluorescence experiments. Each culture dish with permeabilized eSMGs was incubated with 200 µL of reaction cocktail (172 µL of 1× Click-iT^®^ reaction buffer, 8 µL of CuSO_4_, 0.48 µL of Alexa Fluor^®^ azide, and 20 µL of 1× Click-iT^®^ EdU buffer additive) protected from light at RT for 30 min. The eSMGs were washed once with 3% *w*/*v* PBS-BSA, then additional staining with primary and secondary antibodies and image acquisition were performed as described above.

### 4.7. RNA Isolation and RNA-Seq Data Analysis

Up to 10 eSMGs were collected in sterilized tubes with TRIzol^®^ reagent (Invitrogen, 15596026) at 6 h after AMD3100 treatment and were homogenized with SuperFast^®^ Prep-2 (MP Biomedicals, MP116012500; Santa Ana, CA, USA). Total RNA was extracted using the Direct-zol^®^ RNA MiniPrep kit (Zymo Research, R2050; Tustin, CA, USA) according to the manufacturer’s instructions. Library construction (50 bp, single read) and RNA-seq were carried out by Ebiogen, Inc. (Seoul, Korea) using a QuantSeq 3′ mRNA-Seq Library Prep Kit (Lexogen, Greenland, NH, USA) and NextSeq 500 (Illumina, San Diego, CA, USA), respectively. Raw read count data were normalized using the edgeR package in R by the quantile normalization method. The normalized dataset was then unlogged, and fold changes were calculated. *p*-values were determined with R script using the two-sample *t*-test (equal variance) method. R package enrichR was used to perform KEGG pathway enrichment analysis. All analyses and plots were performed and scripted in R (version 4.0.3).

### 4.8. qRT-PCR

Total RNA of the eSMGs was extracted as indicated above. Equal amounts (1 µg) of isolated RNA samples were reverse transcribed using oligo(d)T priming and Superscript IV reverse transcriptase (Invitrogen, 18090010). qRT-PCR reactions were performed with SYBR green using a StepOne^®^ real-time PCR apparatus (Applied Biosystems, 4376357; Foster City, CA, USA). The uniformly expressed control gene *ribosomal protein s32* (*Rps32*) gene served as the control. The amplification protocol was as follows: initial denaturation at 95 °C for 5 min, followed by 40 cycles of denaturation at 95 °C for 5 s, annealing at 60 °C for 30 s, and a final extension at 60 °C for 30 s. Normalized results were then calculated by the comparative *C_t_* (ΔΔ*C_t_*) method. The primer sequences used in this experiment are listed in Table S1.

### 4.9. DNA Extraction and Methyl-Seq

Genomic DNA from eSMGs was isolated with DNeasy^®^ Blood & Tissue Kit (Qiagen, 69504; Valencia, CA, USA). Equal amounts (1 µg) of dsDNA was quantified by absorbance using Qubit^®^ 2.0 fluorometer with dsDNA HS Assay Kit (ThermoFisher). Library preparation (paired-end) and Methyl-seq were carried out by DNALink, Inc. (Seoul, Korea) using the SeureSelectXT^®^ mouse Methyl-seq kit (Agilent Technologies, Santa Clara, CA, USA) and Novaseq 6000 (Illumina), respectively. The mouse mm9 genome build was used as a reference during data analysis. The raw FASTQ files were aligned, and PCR duplicates were discarded using Bismark software (Babraham Bioinformatics, version 0.17.0; Cambridge, UK) without adaptor trimming. For each CpG dinucleotide, the methylated and unmethylated counts and their genomic coordinates were generated as an output file by the bismark_methylation_extractor; the minimum coverage threshold was set at 1. This output file was then processed by Defiant to identify differentially methylated regions.

### 4.10. Statistical Analysis

The results are presented as the mean ± standard error of the mean (SEM) of triplicate independent experiments. Statistical analysis was performed using GraphPad Prism 8 software (GraphPad, La Jolla, CA, USA). The unpaired two-tailed Student’s *t*-test was used to analyze statistical differences between two groups. One-way or two-way analysis of variance (ANOVA) was used to analyze statistical differences between multiple groups using Tukey’s multiple comparison test. *p*-values < 0.05 were considered statistically significant.

### 4.11. Data Deposition

The 3′ mRNA sequencing data is deposited in the Gene Expression Omnibus database (https://www.ncbi.nlm.nih.gov/geo) under accession number GSE160735.

## Data Availability

All relevant data are within the paper and its Supplementary Materials. The RNA-seq data is deposited in the GEO database under accession number GSE160735.

## Supplementary Materials

Supplementary materials can be found at https://www.mdpi.com/1422-0067/22/2/619/s1.
