# Supplementary material for: CXCR4 Regulates Temporal Differentiation via PRC1 Complex in Organogenesis of Epithelial Glands
Source: Int J Mol Sci. 2021 Jan 10;22(2):619. doi: 10.3390/ijms22020619 (PMC7826811; doi:10.3390/ijms22020619)
Supplement: Supplementary file 1 [file ijms-22-00619-s001.pdf]

International Journal of
Molecular Sciences

MDPI

Article

CXCR4 Regulates Temporal Differentiation via PRC1 Complex in Organogenesis of Epithelial Glands

Supplementary Materials:

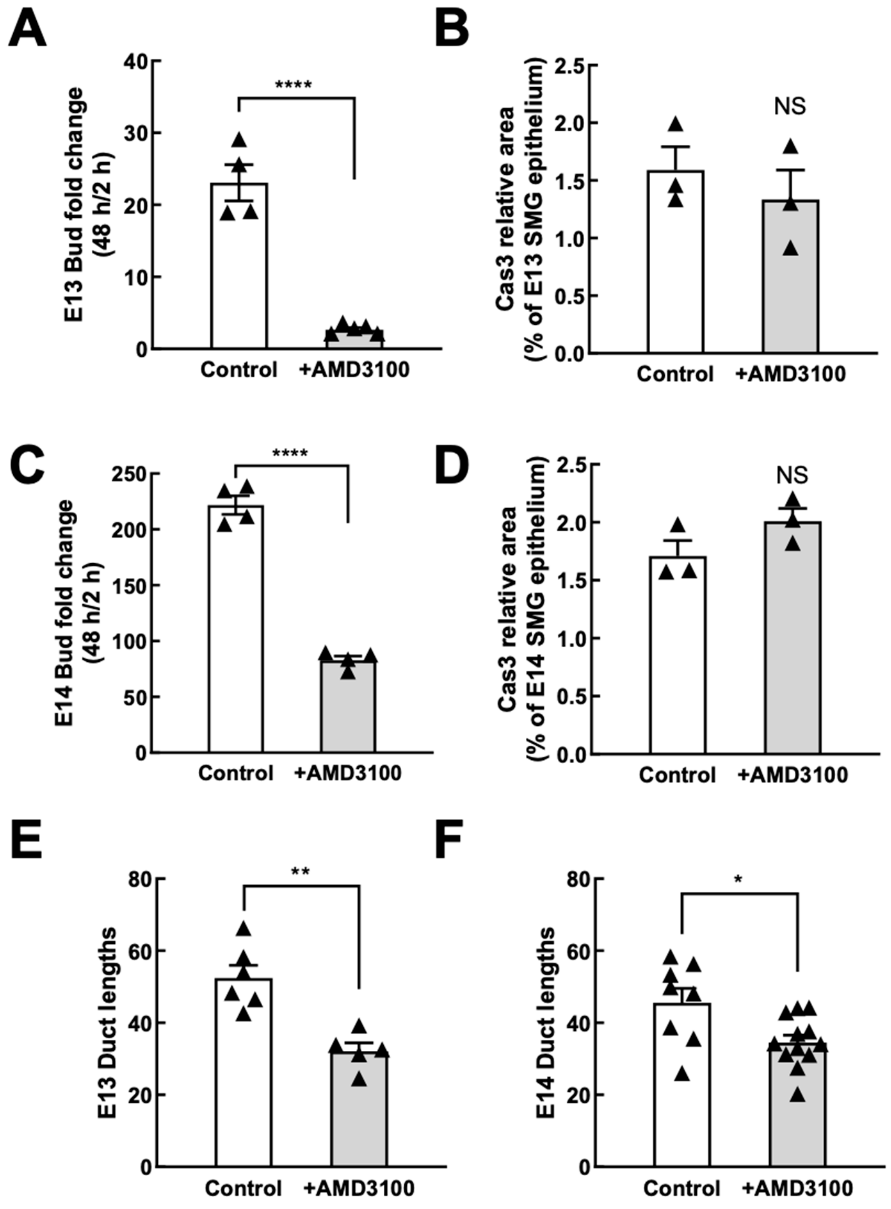

Figure S1. (**A** and **C**) Bud FC over 48 h in E13 (**A**) or E14 (**C**) control and AMD3100-treated eSMGs (n = 4). (**B** and **D**) The relative area (%) of cleaved caspase-3 (Cas3) in the epithelium of E13 (**B**) or E14 (**D**) control and the treated eSMGs (n = 4). (**E** and **F**) The lengths of 3–5 ducts per gland in E13 (**E**) or

E14 (**F**) control and the treated eSMGs are measured and quantified (n = 3). Data are presented as the mean ± SEM; * $p < 0.05$, ** $p < 0.01$, **** $p < 0.0001$, NS: not significant; t-test.

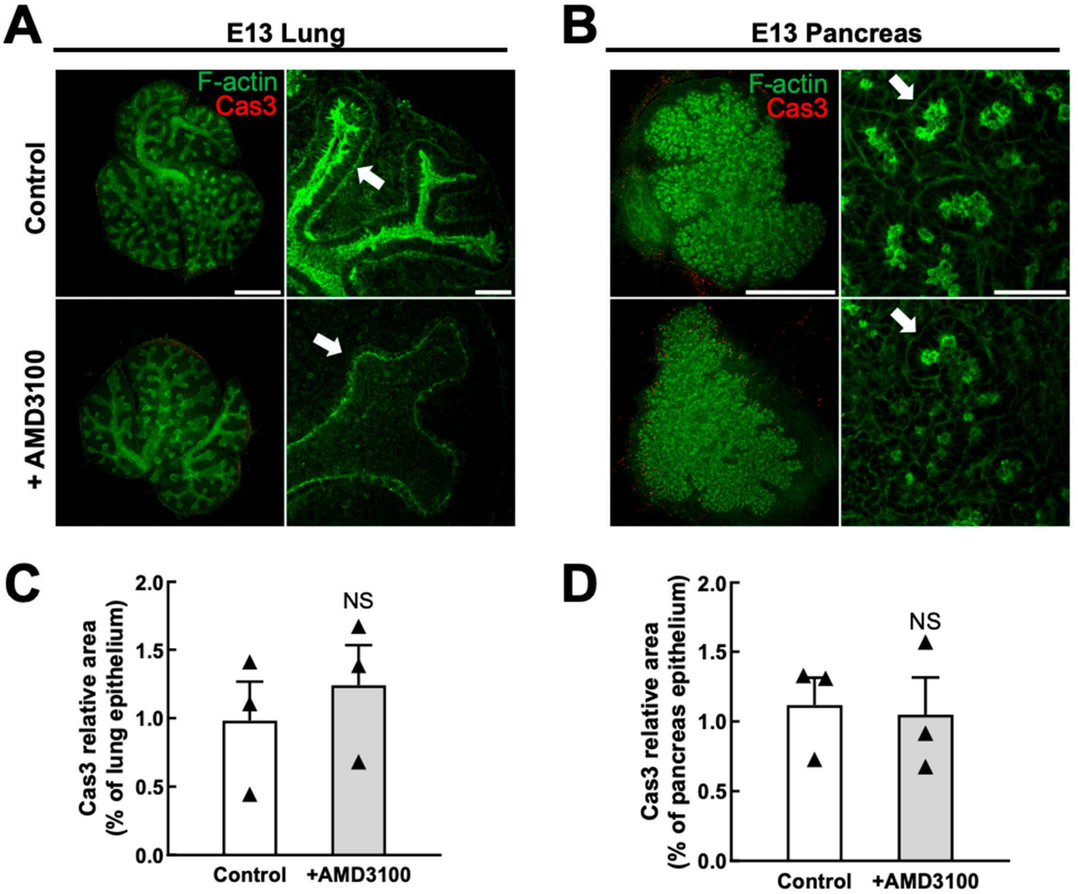

Figure S2. (**A** and **B**) Representative images with F-actin and cleaved caspase-3 (Cas3) in E12 lung (**A**) and pancreas (**B**) (n = 3, scale bars: left panels, 500 μm; right panels, 50 μm). (**C** and **D**) The relative area (%) of cleaved caspase-3 (Cas3) expressions in the epithelium of E12 lung (**C**) and pancreas (**D**) (n = 3). Data are presented as the mean ± SEM; NS: not significant; t-test.

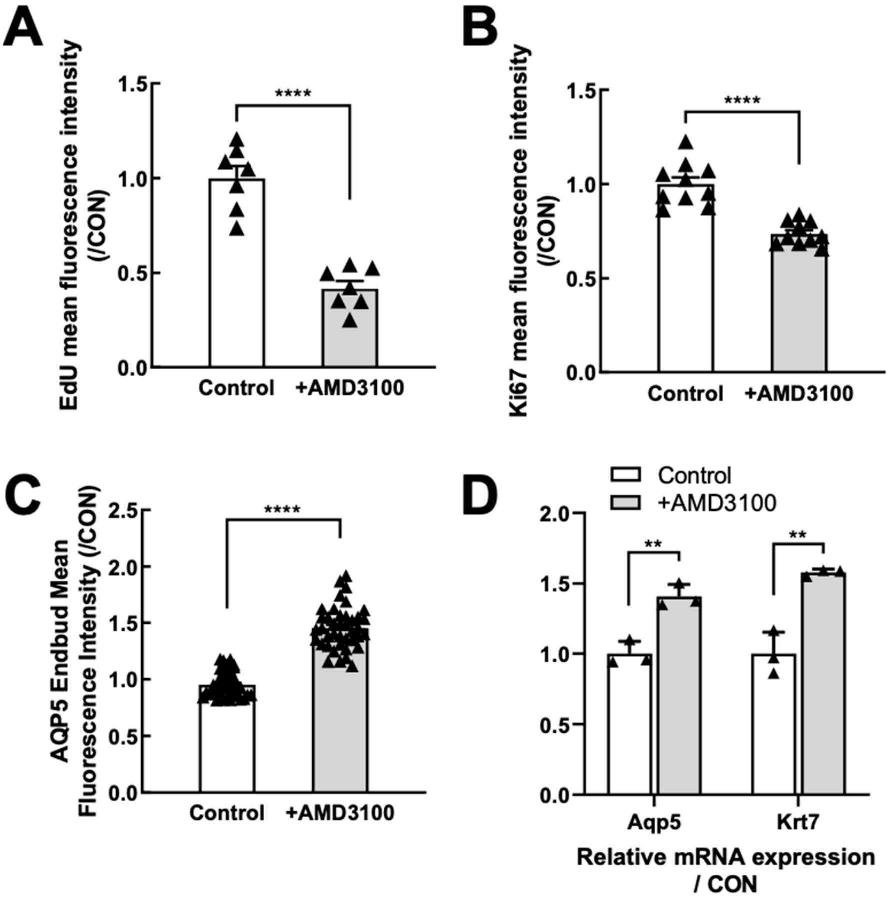

Figure S3. (**A**) Quantification of EdU intensities in control and AMD3100-treated eSMGs (n = 7). (**B**) Quantification of Ki67 intensities in control and AMD3100-treated eSMGs (n = 10). (**C**) Quantification of end bud AQP5 intensities in control and AMD3100-treated eSMGs (n = 3; 10 buds per gland). (**D**) Relative mRNA expression of *Aqp5* and *Krt7* in control and AMD3100-treated eSMGs (n = 3). Data are presented as the mean ± SEM; ** $p < 0.01$, **** $p < 0.0001$; t-test.

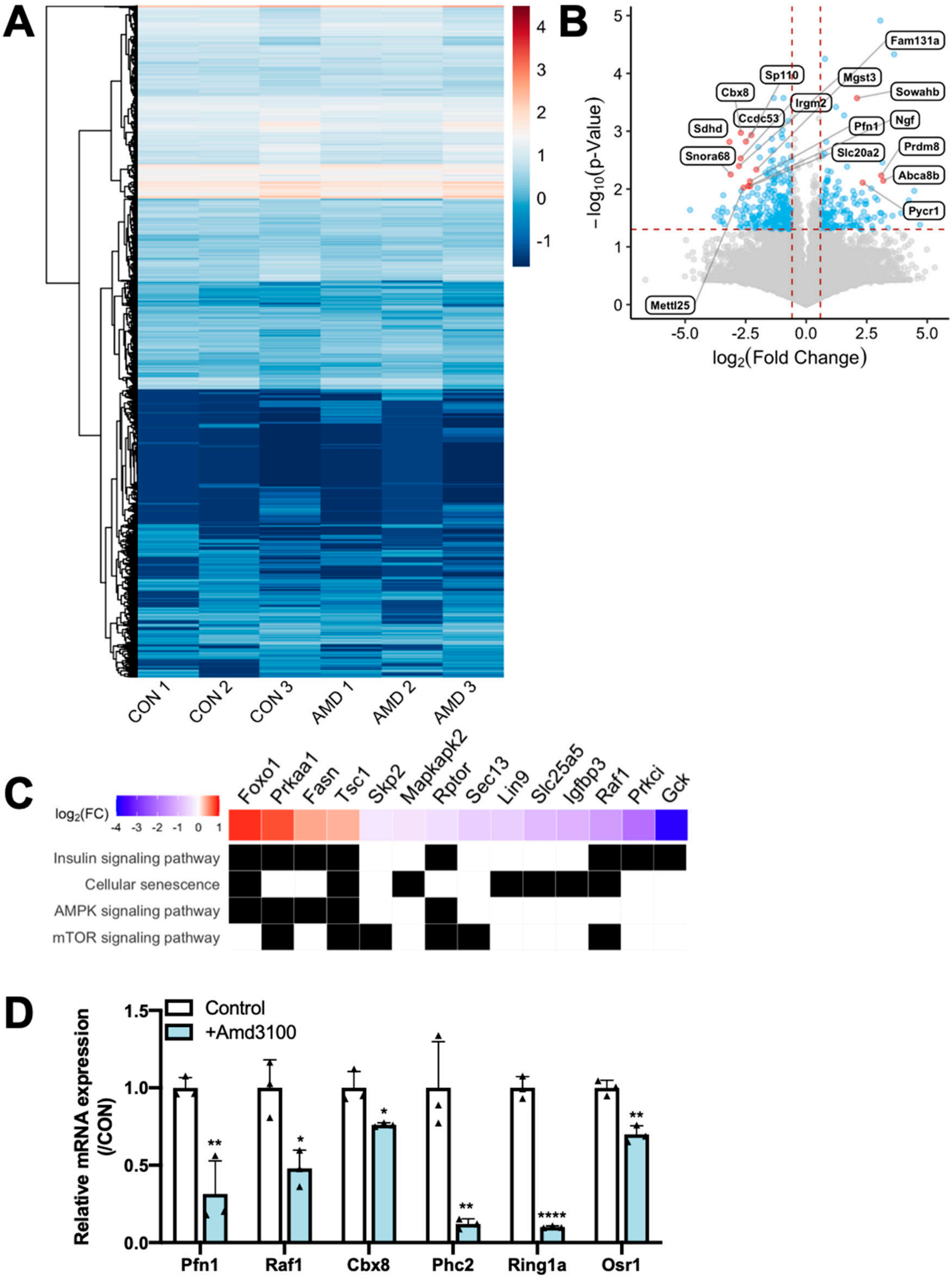

Figure S4. (**A**) Hierarchical clustering heat map of gene expressions (read count *z*-scores) in control and AMD3100-treated eSMGs. (**B**) Volcano scatter plot of DEGs. DEGs with $p < 0.05$ and $|\log2FC| \geq 1$ are in blue and the top DEGs with $p < 0.01$ and $|\log2FC| \geq 2$ are in red. (**C**) DEGs in the four most enriched KEGG pathways (Figure 4B) and their overlapping properties are shown. (**D**) Validation of AMD3100-induced transcriptomic changes with representative DEGs by qPCR. Data are presented as the mean ± SEM; * $p < 0.05$, ** $p < 0.01$, *** $p < 0.001$, **** $p < 0.0001$; *t*-test.

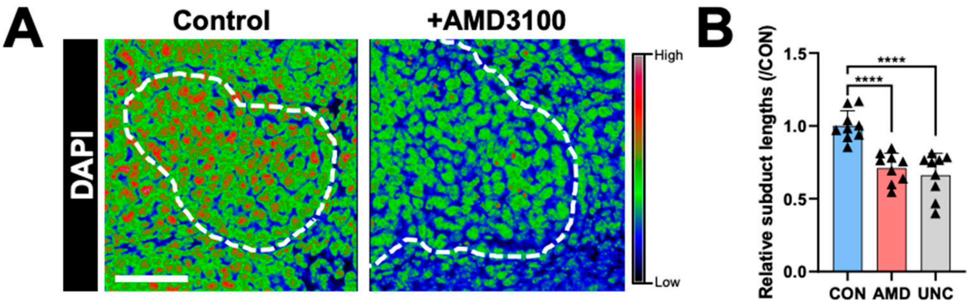

Figure S5. (**A**) Relative color intensity map of DAPI signals in control and AMD3100-treated eSMGs. Colored scale bar indicates (low to high) gray-value of 0–65,535 ($n = 3$, scale bar: 50 μm). (**B**) The mean lengths of the subducts in AMD3100 (AMD)- and UNC3866 (UNC)-treated eSMGs ($n = 9$). Data are presented as the mean ± SEM; **** $p < 0.0001$; one-way ANOVA.

Gene	Forward primer sequence	Reverse primer sequence
Rpl32	5′-TTAAGCGAAACTGGCGGAAAC-3′	5′-TTGTTGCTCCCATAACCGATG-3′
Cxcr4	5′-GAAGTGGGGTCTGGAGACTAT-3′	5′-TTGCCGACTATGCCAGTCAAG-3′
Cxcl12	5′-CAGAGCCAACGTCAAGCA-3′	5′-AGGTACTCTTGGATCCAC-3′
Osr1	5′-TACTCTTTCCTTCAGGCAGTGA-3′	5′-GATCGAGGCAAGTGCATGG-3′
Cdh1	5′-GACTGGAGTGCCACCACCAAAGAC-3′	5′-CGCCTGTGTACCCTCACCATCGG-3′
Krt7	5′-CGCCGCTGAGTGTGGACATCG-3′	5′-CTGGCTGCTCTTGGCTGACTTCTG-3′
Aqp5	5′-TTACTTCTACTTGCTTTTCCCCTCCTC-3′	5′-CGATGGTCTTCTTCCGCTCCTCTC-3′
Krt15	5′-AGGTGGAAGCCGAAGTATCTCT-3′	5′-CATCGCCACCACCAAAATCAC-3′
Pfn1	5′-CTGATGGGCAAAGAAGGTGTC-3′	5′-GGCAGCAATAAGGGAAATGGG-3′
Raf1	5′-TGGACTCAAAGATGCGGTGTT-3′	5′-AAAACCCGGATAGTATTGCTTGT-3′
Cbx8	5′-ATTCGCAAAGGACGCATGGAA-3′	5′-CCTCGCTTTTTGGGGCCATA-3′
Phc2	5′-ATGACCTCAGGGAACGGAAAC-3′	5′-TCGATAACATGCGTCAGGATTTG-3′
Ring1a	5′-CCTGCTTGTGGAGAAAGGAG-3′	5′-ACTGCTTTTCGCTGACACCT-3′

Table S1. Primer sequences used in qPCR experiments.

© 2021 by the author. Licensee MDPI, Basel, Switzerland. This article is an open access article distributed under the terms and conditions of the Creative Commons Attribution (CC BY) license (http://creativecommons.org/licenses/by/4.0/).
